# Structural Characterization of Polysaccharides from Noni (*Morinda citrifolia* L.) Juice and Their Preventive Effect on Oxidative Stress Activity

**DOI:** 10.3390/molecules30051103

**Published:** 2025-02-27

**Authors:** Bin Zhang, Xiaoyu Wei, Peiwen Du, Huangqun Luo, Lanfang Hu, Liping Guan, Guangying Chen

**Affiliations:** 1 School of Food and Pharmacy, Zhejiang Ocean University, Zhoushan 316000, China; x001022y@126.com (X.W.);; 2 Key Laboratory of Tropical Medicinal Resource Chemistry of Ministry of Education, College of Chemistry and Chemical Engineering, Hainan Normal University, Haikou 571158, China

**Keywords:** noni juice, polysaccharides, chemical structure, T2DM, signaling pathway

## Abstract

Polysaccharides are very promising molecules in the field of pharmacotherapy. Knowing this, the aim of this study was to extract, characterize, and evaluate the action of the polysaccharides in noni juice, using biological models of Type 2 diabetes mellitus processes. In this study, one polysaccharide named NJSPd−1 was separated from fermented noni fruit juice. The characterization assay showed that NJSPd−1 had a molecular weight (*Mw*) of 18,545 Da. NJSPd−1 consisted of galacturonic acid, galactose, rhamnose, and arabinose, with a molar ratio of 28.79:20.34:19.80:18.84 according to HPGPC analysis, and the glycosidic bond mainly included →4)-*α*-D-GalA*p*-(1→, 4)-*β*-D-Glc*p*-(1→, →2)-*α*-L-Rha*p*-(1→, and →3)-*α*-L-Ara*f*-(1→. The prevention of oxidative stress activities by NJSPd−1 was evaluated using high-glucose-induced oxidative stress in HepG2 cells. In vitro results showed that NJSPd−1 influenced the downregulation of the proteins and genes Nrf2, Keap1, HO-1, and NQO1 in HepG2 cells. These results suggest that NJSPd−1 exerted a protective effect against oxidative stress in HepG2 cells by activating the Nrf2/HO-1/NQO1 signaling pathway.

## 1. Introduction

Diabetes mellitus (DM), a persistent ailment, stems from disorders in the endocrine metabolism [1]. The International Diabetes Federation (IDF) reported that in 2021, roughly 537 million individuals aged 20 to 79 years suffered from diabetes mellitus. The IDF estimates that this figure will rise to about 643 million by 2030 and further to 783 million by the year 2045 [2]. Type 2 diabetes mellitus (T2DM) is one type of disease that includes severe disorders of the endocrine system and metabolism. The primary indicators of T2DM include insulin resistance (IR), hyperglycemia, and insufficient insulin production [3,4,5]. Insulin is essential for regulating blood glucose levels by triggering responses in peripheral tissues like the adipose tissue, skeletal muscle, and liver. However, the development of insulin resistance can result in serious complications in various organs, including cardiovascular issues and diabetic kidney disease, making it a key contributor to the onset and progression of T2DM [6].

Oxidative stress is a key factor in the progression of IR. Growing evidence suggests that oxidative stress plays a crucial role as an initial trigger in the onset of IR, activating a cascade of pathophysiological signaling pathways that ultimately lead to IR and T2DM. This phenomenon arises when there is an imbalance between the production of malonaldehyde (MDA) and reactive oxygen species (ROS), resulting in cellular damage and dysfunction [7]. However, glutathione peroxidase (GPx), catalase (CAT), and superoxide dismutase (SOD) are crucial antioxidant enzymes that safeguard the body from ROS [8]. Nrf2 moves into the nucleus and associates with the antioxidant response element (ARE). ARE functions as a promoter by attaching to single-stranded DNA, which initiates the transcriptional activation of downstream genes for antioxidant enzymes, including NAD(P)H quinone oxidoreductase 1 (NQO1) and heme oxygenase-1 (HO-1). This pathway can modulate oxidative stress levels [9].

Numerous studies have indicated that various functional food supplements can help mitigate the progression of oxidative stress [10,11]. As a functional food derived from *Morinda citrifolia* L., noni juice has demonstrated multifaceted bioactive properties, including antioxidant effects, hypoglycemic activity, and gut microbiota modulation. Experimental evidence reveals that administration of noni juice effectively ameliorated metabolic dysregulation in murine models, manifesting as significant reductions in blood glucose levels, glycated serum proteins, triglycerides, and low-density lipoprotein cholesterol. Concurrently, it enhanced glucose tolerance and elevated high-density lipoprotein cholesterol, collectively addressing abnormalities in both glycemic control and lipid metabolism. Notably, these therapeutic effects appear to have been context-dependent, as noni juice exhibited no measurable impact on glycemic parameters or glucose homeostasis in normoglycemic rat models [12,13,14]. Additionally, polysaccharides in noni juice have the ability to affect gut microbiota, promote the production of short-chain fatty acids, and reduce colonic barrier permeability as well as metabolic endotoxemia [15]. Noni wine was shown to effectively alleviate oxidative stress and obesity induced by a high-fat diet in mice [16]. However, the mechanism by which noni juice polysaccharide ameliorates oxidative stress has not yet been elucidated. Based on these, this study explored the preventive effects of noni juice polysaccharide in improving oxidative stress via activating the Nrf2/HO-1/NQO1 pathway, hoping to provide new ideas and interventions for the prevention and treatment of diseases related to T2DM.

## 2. Results

### 2.1. Purification of NJSP

**Figure 1:**
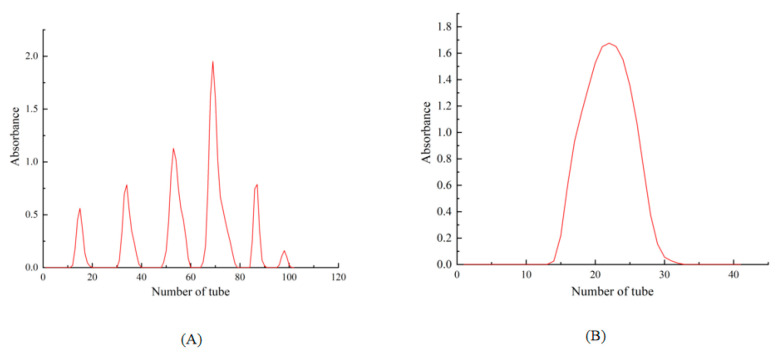
DEAE-52 column chromatography of NJSP (**A**). Sephadex G-75 column chromatography of NJSPd−1 (**B**).

The polysaccharides extracted from noni juice (NJSP) were separated using a DEAE-52 cellulose column to isolate the primary component NJSPd (Figure 1A). This was further purified using a Sephadex G-75 column, resulting in the isolation of the purified polysaccharide, which was named NJSPd−1 (Figure 1B).

### 2.2. Characterization of NJSPd−1

#### 2.2.1. Analysis of Molecular Weight and Monosaccharide Composition of Noni Juice Polysaccharide

**Figure 2:**
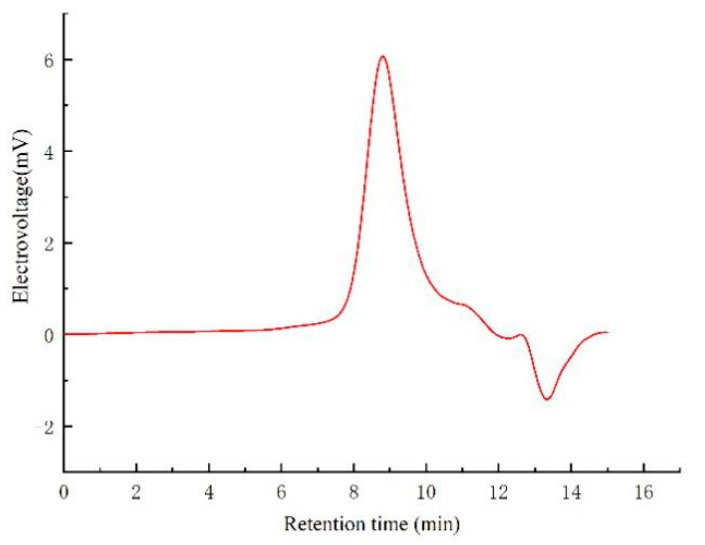
HPGPC chromatogram of NJSPd−1.

HPGPC was an effective method for the *M_w_* determination of the polysaccharide. As shown in Figure 2, the elution profile displayed a single, symmetrical peak, suggesting that NJSPd−1 contained only one polysaccharide fraction. Based on a retention time of 18.57 min, the molecular weight was calculated to be 18,545 Da. Compared with the noni polysaccharide, NJSPd−1 exhibited a lower molecular weight, potentially leading to enhanced bioactivity [17].

The monosaccharide composition of the polysaccharide was examined through HPLC analysis (Table 1). NJSPd−1 primarily consisted of galacturonic acid, galactose, rhamnose, and arabinose, in a molar ratio of 28.79:20.34:19.80:18.84. These findings clearly indicate that NJSPd−1 was an acid heteropolysaccharide composed of galacturonic acid [18].

#### 2.2.2. FT-IR Spectrum Analysis of Noni Juice Polysaccharide

**Figure 3:**
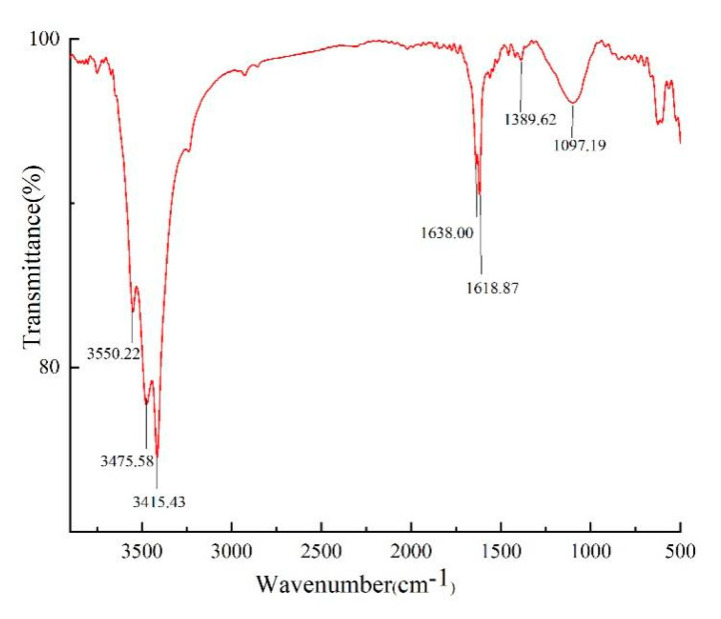
FT-IR spectra of NJSPd−1 sample.

NJSPd−1 was characterized by FT-IR, as shown in Figure 3. Typical signals of polysaccharide at frequencies around 3550, 3475, 3415, 1638, 1618, 1389, and 1097 cm^−1^ were clear for the NJSPd−1 samples. Broad bands at 3550, 3475, and 3415 cm^−1^ indicated the presence of intermolecular or intramolecular −OH. The peaks at 1638, 1618, and 1389 cm^−1^ were attributed to the asymmetric and symmetric stretching of −COOH, suggesting the presence of uronic acid. The NPSPd-1 samples showed strong absorption peakbands in the region of 1097 cm^−1^, which indicated that this polysaccharide contained a C-O-C glycosidic bond and pyranose ring structure.

#### 2.2.3. NMR Spectrum Analysis

**Figure 4:**
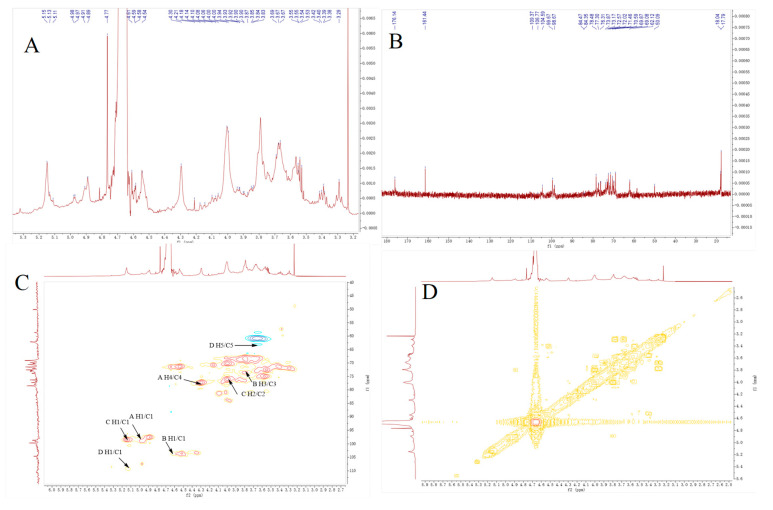
NMR analysis of NJSPd−1. (**A**) ^1^H NMR spectrum; (**B**) ^13^C NMR spectrum; (**C**) ^1^H-^1^H COSY NMR spectrum; (**D**) ^1^H-^13^C HSQC NMR spectrum.

As shown in Figure 4A,B, the characteristic resonance signals of anomeric protons were detected at *δ*_H_ 4.0–5.3 ppm, while those of anomeric carbon were found at *δ*_C_ 90–110 ppm [19]. Four chemical shifts of *δ*_H_/*δ*_C_ 4.98/99.67, 4.62/104.59, 5.15/98.67, and 5.11/109.34 were attributed to the heterotopic signal of H1/C1 in NJSPd−1. Moreover, the signals at *δ*_H_ 3.1–4.2 and *δ*_C_ 60–90 represented the non-anomeric atoms of H2–H6 and C2–C6, respectively. The four main sugar residues may have been as follows: A: *α*-D-galacturonic acid, B: *β*-D-galactose, C: *α*-L-rhamnose, and D: *α*-L-arabinose [20,21,22,23,24]. These findings are consistent with the FT-IR data. To obtain detailed structural information, COSY and HSQC were analyzed (Figure 4C,D). The signals of H1/C1 (*δ* 4.98/99.67), H2 (*δ* 3.82), H3 (*δ* 4.03), H4/C4 (*δ* 4.30/77.30), H5 (*δ* 4.61), and a carbon signal at *δ_C_* 176.14 indicated that A →4)-*α*-D-GalA*p*-(1→ was detected [25,26]. The presence of a residue B →4)-*β*-D-Gal*p*-(1→ unit was indicated based on at H1/C1 (*δ* 4.62/104.59), H3/C3 (*δ* 3.67/73.87) [27]. Chemical shifts of H1/C1 (*δ* 5.15/98.67), H2/C2 (*δ* 4.06/76.31), H3 (*δ* 4.01), H4 (*δ* 3.29), and H5 (*δ* 3.72) were assigned to the sugar residue C →2)-*α*-L-Rha*p*-(1→ [28]. In addition, the signals observed at H1/C1 (*δ* 5.11/109.37) and H5/C5 (*δ* 3.66/62.12) suggested the presence of D →3)-*α*-L-Ara*f*-(1→ [29]. The resonance signal of ^13^C NMR at *δ*_C_ 176.14 belonged to the anomeric carbon atom of the acetyl group, meaning the O-2 or O-3 position of the methylated →4)-*α*-GalAp(1→ may have been substituted by an acetyl group.

The results presented above indicate that the NJSPd−1 structure mainly consisted of a linear backbone made up of *O*-acetylated-(1→4)-linked-*α*-GalA*p* along with a small fraction of (1→2)-Rha*p*. Additionally, there was a branch at C-4 that included a backbone of (1→4)-linked-*β*-Gal*p*, as well as a branch containing (1→3)-linked-α-Ara*f* [17]. These observations align with earlier studies.

### 2.3. Effect of Different Concentrations of NJSPd−1 on the Viability of HepG2 Cells

**Figure 5:**
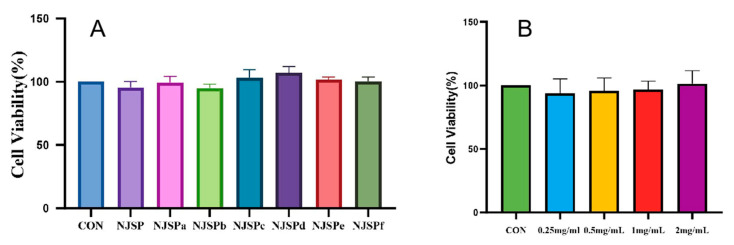
Effect of different doses of polysaccharides from noni juice on the viability of HepG2 cells. (**A**) Cell viability of NJSP and NJSPa-f; (**B**) Cell viability of NJSPd-1. All values are expressed as means ± SD, *n* = 3.

The CCK8 method was used to detect the viability of HepG2 cells following treatment with NJSPa-f. As shown in Figure 5A, NJSP had no significant effect on cytotoxicity compared with the normal control at 48 h. Meanwhile, NJSPd showed the highest cellular activity. To identify the ideal concentration of NJSPd−1 for experiments involving HepG2 cells, we first assessed cell viability using the CCK8 method across a range of NJSPd−1 concentrations (0.25–2.0 mg/mL). As shown in Figure 5B, the cell viability in all groups exhibited no significant decrease compared with the normal control group, which was treated with various concentrations of NJSPd−1 for 24 h. The CCK8 result showed that the HepG2 cell viability exhibited no obvious toxicity following treatment with 0.25–2.0 mg/mL of NJSPd−1. Therefore, 0.5, 1.0, and 2.0 mg/mL of NJSPd−1 were employed for subsequent study.

### 2.4. Effects of NJSPd−1 on Oxidative Stress in HepG2 Cells

**Figure 6:**
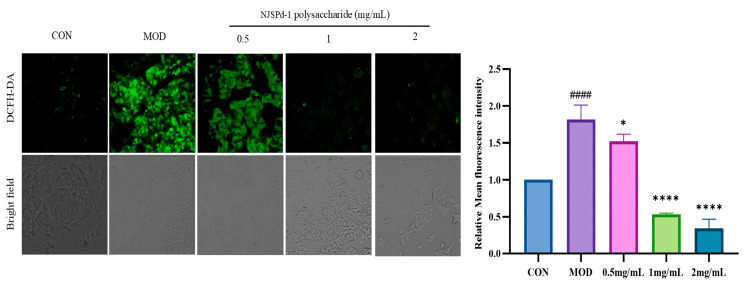
Effects of different doses of NJSPd−1 on the relative mean fluorescence intensity of ROS of HepG2 cells. All values are expressed as means ± SD, *n* = 3. **** *p* < 0.0001 and * *p* < 0.05 compared with CON group. ^####^ *p* < 0.0001 compared with MOD group.

Following the treatment, the intracellular oxidative stress levels were assessed based on fluorescence intensity. Increase in the intensity of intracellular fluorescence was used as an indicator of elevated intracellular ROS activity. The photomicrograph of stained HepG2 cells in Figure 6 illustrates the impact of high glucose on the generation of intracellular ROS. The accumulation of intracellular ROS meant that after treatment with 0.5–2.0 mg/mL of NJSPd−1, the DCFH-DA-stained HepG2 cells exhibited reduced fluorescence. Treatment with NJSPd−1 resulted in a decrease in intracellular ROS activity in a dose-dependent manner, compared with the model control (*p* < 0.05). Furthermore, the data indicated that NJSPd−1 treatment led to a reduction in ROS production, possibly contributing to its preventive effects against oxidative stress through multiple pathways.

### 2.5. Western Blot Analysis

**Figure 7:**
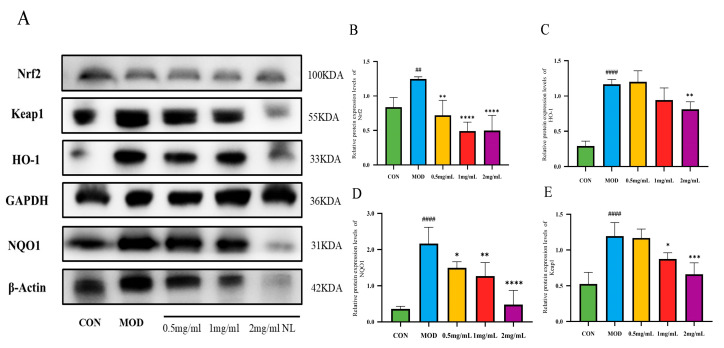
Relative protein expression levels. (**A**) Protein expression levels; (**B**) Nrf2; (**C**) HO-1; (**D**) NQO1; (**E**) Keap1. All values are expressed as means ± SD, *n* = 3. * *p* < 0.05, ** *p* < 0.01, *** *p* < 0.001 and **** *p* < 0.0001 compared with CON group. ^####^ *p* < 0.0001 and ^##^ *p* < 0.01 compared with MOD group.

In the Western blot analysis, NQO1 levels in HepG2 cells were normalized to the *β*-actin bands, while the levels of other proteins were normalized to the GAPDH bands. In our study, the expression levels of the Nrf2 protein in the MOD group were significantly increased (*p* < 0.01) compared with the CON group. Meanwhile, the groups receiving NJSPd−1 (0.5–2.0 mg/mL) treatment exhibited a reduction in Nrf2 expression (*p* < 0.01), as shown in Figure 7A,B. Additionally, a dose–response relationship was observed. The results showed that HepG2 cells had enhanced antioxidant stress ability after treatment with NJSPd−1. In a similar manner, different concentrations of NJSPd−1 demonstrated a notable regulatory impact on key proteins within the Nrf2/HO-1/NQO1 signaling pathway. The expression levels of HO-1, Keap1, and NQO1 proteins in the NJSPd−1 group were significantly lower (*p* < 0.05) compared with those in the MOD group (Figure 7). This indicates that NJSPd−1 may have increased the anti-oxidative stress response in HepG2 cells by modulating the Nrf2/HO-1/NQO1 signaling pathway.

### 2.6. qPCR Analysis

**Figure 8:**
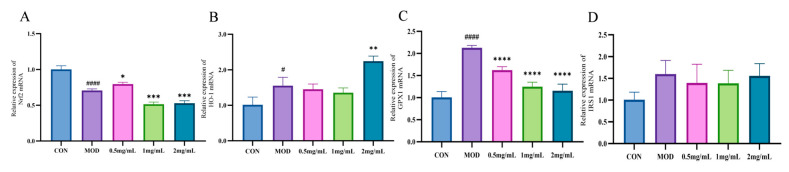
Relative expression of mRNA. (**A**) Nrf2; (**B**) HO-1; (**C**) GPX1; (**D**) IRS1. All values are expressed as means ± SD, *n* = 3. * *p* < 0.05, ** *p* < 0.01, *** *p* < 0.001 and **** *p* < 0.0001 compared with CON group. ^####^ *p* < 0.0001 and ^#^ *p* < 0.05 compared with MOD group.

GAPDH served as the internal reference gene, and mRNA expression levels in the CON group were used to normalize those of the other groups. The protein and mRNA expression levels of Nrf2/HO-1/NQO1 pathway-related factors in HepG2 cells in each group are shown in Figure 8. The levels of Nrf2, HO-1, GPX1, and IRS1 in the three NJSPd−1 dosage groups (0.5–2.0 mg/mL) were significantly lower than those in the MOD group (*p* < 0.05), indicating that NJSPd−1 enhanced antioxidant activity. The results showed that NJSPd−1 was able to enhance the antioxidant stress effect by regulating the Nrf2/HO-1/NQO1 signaling pathway in HepG2 cells.

## 3. Discussion

The antioxidant mechanism of *Morinda citrifolia* (noni) polysaccharides has long been overshadowed by research focusing on its small-molecule components, such as iridoids and phenolic acids, which typically activate the Nrf2/ARE pathway to combat oxidative stress [30]. In contrast, our findings reveal that NJSPd−1, a novel polysaccharide from fermented noni juice, paradoxically downregulated Nrf2, HO-1, and Keap1 in high-glucose-exposed HepG2 cells (Figure 7 and Figure 8). This divergence challenges the canonical paradigm of Nrf2 activation as the primary antioxidant mechanism in noni-derived compounds. For instance, Zhang et al. (2020) demonstrated that noni fruit extract upregulated Nrf2 to reduce hepatic oxidative damage in diabetic rats [31]. The suppressive effect of NJSPd−1 on Nrf2 highlights a context-dependent regulatory strategy, potentially avoiding the deleterious consequences of chronic Nrf2 overactivation.

Structurally, NJSPd−1’s low molecular weight (18.5 kDa) and acetylated galacturonic acid backbone (Figure 4) may explain its unique bioactivity. NJSPd−1 has a high content of galacturonic acid, and its carboxyl group may play an antioxidant role by directly scavenging free radicals or chelating metal ions [32,33]. The current study revealed that NJSPd−1 alleviates oxidative stress by downregulating the expression of Nrf2, HO-1, NQO1 and other proteins, which is different from the mechanism of direct activation of the Nrf2 pathway by traditional antioxidants (such as vitamin C). It is speculated that NJSPd−1 may restore REDOX homeostasis by inhibiting hyperglycemia-induced Nrf2 overactivation, thereby avoiding cell damage caused by long-term stress [34]. This “adaptive regulation” mechanism provides a new perspective for targeted intervention with natural polysaccharides. NJSPd−1’s compact structure probably facilitates direct interaction with Keap1, modulating redox homeostasis without triggering pathway hyperactivity. Xu et al. (2020) reported that high-dose Se alleviated pancreatic injury via Nrf2/NQO1/HO-1 upregulation in acute pancreatitis models [18], whereas NJSPd−1’s suppression of Nrf2 under high glucose suggests a tailored mechanism for chronic metabolic disorders.

Critically, NJSPd−1’s high galacturonic acid content (28.79%, Table 1) may enhance its solubility and stability, positioning it as a viable functional food additive. However, our reliance on an in vitro HepG2 model raises translational concerns. While in vivo studies confirm noni’s systemic benefits, such as gut microbiota modulation and lipid profile improvement, NJSPd−1’s tissue-specific effects remain unverified. This discrepancy underscores the need for in vivo validation to reconcile mechanistic insights with physiological outcomes.

However, there are still many limitations to this paper, such as the use of in vitro cell models that cannot fully simulate the complex metabolic environment of organisms. It is recommended to use diabetic animal models (e.g., db/db mice) to verify the hypoglycemic, insulin resistance improvement, and organ protection effects of NJSPd−1 and to evaluate its pharmacokinetic characteristics (e.g., bioavailability, tissue distribution). The interaction with intestinal flora (such as short-chain fatty acid production) can be further explored to expand its application in metabolic regulation.

## 4. Materials and Methods

### 4.1. Materials

The noni juice used for this investigation was collected from Songji Yunshang Technology Limited Company (Hainan, China) in October 2020. Dextran standards, monosaccharide standards, and 1-phenyl-3-methyl-5-pyrazolone (PMP) were obtained from Sigma-Aldrich Chemical Co. (St. Louis, MO, USA). The dialysis bag with a molecular weight cutoff of 3500 Da was sourced from Union Carbide Co. (Viskase, Lombard, IL, USA). Dulbecco’s modified Eagle’s medium (DMEM), penicillin–streptomycin solution, and fetal bovine serum (FBS) were purchased from Gibco-BRL (Grand Island, NY, USA). The Cell Counting Kit-8 (CCK8) was purchased from ApexBio (Houston, TX, USA). HepG2 cells were obtained from ZQXZBIO (Shanghai, China). Ethanol and other solvents were analytical grade and purchased from Sinopharm Chemical Reagent Co., Ltd. (Shanghai, China). All salts were analytical grade and purchased from Aladdin Biotechnology Co., Ltd. (Shanghai, China).

### 4.2. Extraction

Noni juice was extracted at 25 °C for 8 h using petroleum ether to eliminate lipids [35]. Subsequently, four volumes of 95% ethanol were added, and the mixture was stored at 4 °C for a duration of 12 h. Filtration was then performed to collect the precipitate. The crude polysaccharide from the noni juice was obtained through lyophilization. The polysaccharide content was measured using the phenol–sulfuric acid method [36], and the yield of polysaccharide was calculated using the following equation:

$$\mathrm{yield}\;(\%)=\frac{\mathrm{Crudepoly}\;\mathrm{saccharide}\;\mathrm{weight}\;(g)}{\mathrm{Raw}\;\mathrm{material}\;\mathrm{weight}\;(g)}\;\times \;100\%$$

### 4.3. Purification of Crude Polysaccharide

The crude NJSP was purified using a DEAE-52 cellulose chromatographic column. The elution process involved the initial use of water, followed by sodium chloride solutions at concentrations of 0.1, 0.2, 0.3, 0.4, and 0.5 M, with a flow rate of 1 mL/min [37]. The collected eluates were combined based on their total carbohydrate content, which was measured using the phenol–sulfuric acid method, resulting in six fractions: NJSPa, NJSPb, NJSPc, NJSPd, NJSPe, and NJSPf. The NJSPd fraction underwent further purification using a Sephadex G-100 column, eluted with a 0.3 M NaCl solution, and the main eluting peaks were collected. NJSPd−1 was subsequently acquired by means of vacuum concentration and freeze-drying following dialysis [38].

### 4.4. Characterization of NJSPd−1

#### 4.4.1. Homogeneity and Molecular Weight

The molecular weight and purity of NJSPd−1 were evaluated through high-performance gel permeation chromatography. The molecular weight of NJSPd−1 was determined using a calibration curve based on T-series Dextran standards with molecular weights of 330, 176, 82.5, 44, 25.3, 20.6, 12.6, 7.13, 4.29, and 1.4 kDa.

#### 4.4.2. Analysis of Monosaccharide Composition

The monosaccharide composition of NJSPd−1 was examined using HPLC [39]. A weighed 20 mg sample of NJSPd−1 was hydrolyzed using 5 mL of 2 mol/L TFA at 120 °C for 2 h. After removing the TFA with methanol, the vacuum-dried monosaccharide hydrolyzate was re-dissolved in 5 mL of distilled water. The standard monosaccharide mixed solution was then analyzed by HPLC following the specified method. The mobile phase consisted of 50 mM ammonium acetate and 100% acetonitrile, maintained at a temperature of 30 °C, with a regulated flow rate of 0.4 mL/min.

#### 4.4.3. FT-IR Spectrometric Analysis

IR spectra were obtained using the KBr-disk technique with a Fourier transform infrared (FT-IR) spectrometer (Nicolet 6700, Thermo Scientific, Waltham, MA, USA) over a range of 500–4000 cm^−1^.

#### 4.4.4. NMR Spectrometric Analysis

NMR spectra of the NJSPd−1 samples were recorded with a JNM-ECZ600R (JEOL, Tokyo, Japan). For this purpose, 25 mg of NJSPd−1 was dissolved in D_2_O, and the ^1^H NMR and the ^13^C NMR spectra were recorded at 25 °C [40].

### 4.5. Cell Treatment and Grouping

HepG2 cells were sourced from ZQXZBIO in Shanghai, China. These cells were cultured in DMEM enriched with 1.5 g/L NaHCO_3_, 110 mg/L sodium pyruvate, 100 U/mL penicillin, 100 μg/mL streptomycin, and 10% FBS and were kept at 37 °C in a humidified incubator with 5% CO_2_.

HepG2 cells were seeded in 6-well culture plates and permitted to adhere at 37 °C for 24 h. The cells were then incubated with NJSPd−1 (0.5, 1.0, and 2.0 mg/mL) for 2 mL at 37 °C for an additional 2 h. Subsequently, 2 mL of 50 mM high-glucose complete medium was added to the NJSPd−1 group and the model control, with the normal control treated with regular glucose (5.5 mM) at 37 °C for an additional 48 h. Western blotting and qPCR were employed to detect the key enzymes in the Nrf2/HO-1/NQO1 pathway to elucidate the molecular underpinnings of fermented noni juice polysaccharides on the oxidative stress prevention effects.

### 4.6. Cell Activity Assay

Cell viability was evaluated using a cell counting kit-8 (CCK-8) assay [41]. In summary, HepG2 cells were cultured in a 96-well plate at a density of 1 × 10^5^ cells per well and treated with varying concentrations of NJSPd−1 for a duration of 48 h. Absorbance was measured with a microplate reader at 450 nm wavelength, and control cells (CON) were considered to have 100% cell viability. The experimental concentration of NJSPd−1 was determined based on the results of this study.

### 4.7. ROS Detection

Intracellular levels of ROS were assessed using DCFH-DA in accordance with the instructions from the ROS Assay Kit manufacturer. HepG2 cells were treated with DCFH-DA for 35 min at 35 °C. After incubation, the cells were analyzed using an inverted fluorescence microscope from Olympus Corporation (ckx 53, Tokyo, Japan). Each experiment was conducted in triplicate [42].

### 4.8. Western Blot Method

Western blot analysis was performed using total, membrane, and nuclear proteins extracted from HepG2 cells. Equal quantities of protein (100 μg per sample) were subjected to electrophoresis using 12% SDS-PAGE, followed by transfer to PVDF membrane. The membranes were incubated in a blocking solution of 5% non-fat milk for 1 h, followed by treatment with the appropriate primary antibodies at the specified dilution ratio, continuing to incubate overnight at 4 °C. The membranes were subsequently incubated with the specific secondary antibody for 1.5 h at room temperature. Finally, the antigen–antibody complexes were identified using the ECL chemiluminescence kit. Densitometric analysis was conducted using ImageJ software (1.8.0.112) [43].

### 4.9. qPCR Analysis

HepG2 cells were plated at a density of 5 × 10^5^ cells per well in a 6-well plate and incubated for 24 h. Following treatment with NJSPd−1, total RNA extraction from cells was carried out employing an Eastep^®^ super total RNA extraction kit (Shanghai Promega, Shanghai, China) according to the manufacturer’s instructions. Subsequently, cDNA was synthesized from 500 ng of total RNA with Hifair^®^ III 1st Strand cDNA Synthesis SuperMix (Yeasen, Shanghai, China) which was then subjected to qPCR using an SYBR Green Kit on a CFX96 system. The reaction conditions were as follows. The hot-start DNA polymerase was activated at 95 °C for 8 min, followed by 40 cycles of denaturation at 95 °C for 10 s, and primer annealing/extension at 58 °C for 1 min [44]. The relative density of mRNA expression was normalized against the internal reference gene GAPDH and subsequently analyzed using the 2^−ΔΔCt^ method. All primer sequences of Nrf2, IRS1, HO-1, GXP1 for qPCR are listed in Table 2.

### 4.10. Statistical Analysis

All data are represented as mean ± standard deviation (SD). One-way ANOVA was employed for statistical comparisons between groups, with the analyses conducted via GraphPad Prism 10. *p* < 0.05 was statistically meaningful and reliable.

## 5. Conclusions

*Morinda citrifolia* (noni) is rich in various bioactive compounds that provide several health benefits. These include antioxidant effects, the regulation of blood glucose and lipid metabolism, as well as anti-cancer, anti-inflammatory, and antibacterial properties. Noni and its by-products have strong antioxidant activity. The water extract of noni fruit and noni fruit polysaccharide were found to reduce hepatic malondialdehyde levels and increase hepatic Trolox-equivalent antioxidant capacity levels. Noni and noni juice have the potential to alleviate oxidative stress, ameliorate insulin resistance, and help regulate the gut microbiome in diabetes. Incorporating fermented noni fruit powder into the diets of mice with T2DM enhanced insulin sensitivity and lowered levels of triglycerides, low-density lipoprotein cholesterol, and glycosylated hemoglobin. Noni juice exerts hypoglycemic effects and improves diabetes and oxidative stress by activating the Nrf2/ARE pathway. These results indicate that noni and its by-products provide good antioxidant effects and can improve the body’s resistance to oxidative stress. However, the polysaccharides of fermented juice from noni fruits have not been studied. The current results showed that NJSPd−1 significantly increased cell viability and attenuated ROS production in HepG2 cells. Pre-treatment with NJSPd−1 may help combat antioxidant stress by affecting the levels of Nrf2, HO-1, Keap1, and NQO1. Therefore, noni juice polysaccharide can help prevent T2DM via enhancing the antioxidant stress response.

Our research examined the fine structure of a polysaccharide (NJSPd−1) isolated from noni juice. NJSPd−1 was characterized as a homogeneous heteropolysaccharide composed of galacturonic acid, galactose, rhamnose, and arabinose with a molar fraction of 28.79%, 20.34%, 19.80%, and 18.84%. There were four main sugar residues in NJSPd−1, including →4)-*α*-D-GalA*p*-(1→, 4)-*β*-D-Glc*p*-(1→, →2)-*α*-L-Rha*p*-(1→, and →3)-*α*-L-Ara*f*-(1→. In this study, an oxidative stress model of HepG2 cells was success fully established by exposure to 50mM high glucose complete medium and the effects of NJSPd−1 pretreatments at different concentrations of NJSPd−1 (0.5, 1.0, and 2.0 mg/mL) on HepG2 cells were evaluated. Through the HepG2 cell model induced by high glucose in vitro, this study demonstrated for the first time that NJSPd−1 significantly inhibited ROS production and reduced oxidative stress by regulating the Nrf2/HO-1/NQO1 signaling pathway in a dose-dependent manner. This finding provides an important theoretical basis for the functional application of noni polysaccharide in diabetes mellitus and related metabolic diseases. As a traditional functional food, Noni juice has low cytotoxicity (0.25–2.0 mg/mL without significant inhibition) and the high antioxidant activity of its polysaccharide component NJSPd−1 makes it suitable for development as an adjuvant therapeutic agent or functional food additive for diabetes. In addition, its structural properties (such as acidic groups) may give it good water solubility and stability, conducive to industrial production.

As an active polysaccharide in noni juice, NJSPd−1’s unique structural characteristics and antioxidant mechanism provide a new idea for the development of natural products. Future research needs to be combined with multidisciplinary techniques (e.g., synthetic biology, nanodelivery systems) to optimize its functional properties and promote the transition from basic research to clinical application. The further development of noni polysaccharides is expected not only to provide innovative strategies for diabetes management, but also to open up potential pathways for intervention in other oxidative stress-related diseases such as cardiovascular disease and neurodegenerative diseases. The current results indicate that NJSPd−1 may have potential value in the pharmaceutical and functional food industries.

## Figures and Tables

**Table 1:** Monosaccharide components (mol%) of NJSPd−1 samples obtained.

Sample	ManA	GluA	GalA	Man	Glu	Gal	Rha	Fuc	Rib	Xyl	Ara
NJSPd−1	0.49	0.88	28.79	0.18	0.64	20.35	19.80	3.50	0.78	5.70	18.84

**Table 2:** Primers used for qPCR.

Gene	Forward Primer Sequence (5′-3′)	Reverse Primer Sequence (5′-3′)
Nrf2	CAGTCAGCGACGGAAAGAGTA	TGTGGGCAACCTGGGAGTAG
HO-1	TGGCTGGCTTCCTTACCGT	ACCACCCCAACCCTGCTAT
IRS-1	GGGAGGACTTGAGCTACGGT	GATGGGGTTAGAGCAGTTGGA
GXP1	GCAACCAGTTTGGGCATCA	CCGTTCACCTCGCACTTCTC

## Data Availability

Data are contained within the article and Supplementary Materials.

## Supplementary Materials

The following supporting information can be downloaded at: https://www.mdpi.com/article/10.3390/molecules30051103/s1, Figure S1. ^1^H NMR spectrum of NJSPd−1. Figure S2. ^13^C NMR spectrum of NJSPd−1. Figure S3. HSQC spectrum of NJSPd−1. Figure S4. ^1^H-^1^H COSY spectrum of NJSPd−1.
