# Supplementary material for: Structural Characterization of Polysaccharides from Noni (Morinda citrifolia L.) Juice and Their Preventive Effect on Oxidative Stress Activity
Source: Molecules. 2025 Feb 27;30(5):1103. doi: 10.3390/molecules30051103 (PMC11902223; doi:10.3390/molecules30051103)
Supplement: Supplementary file 1 [file molecules-30-01103-s001.zip › molecules-3494130-supplementary.pdf]

Structural Characterization of Polysaccharides from Noni (*Morinda citrifolia* L.) Juice and Their Preventive Effect on Oxidative Stress Activity

Bin Zhang [1,*,†], Xiaoyu Wei [1,†], Peiwen Du [1], Huangqun Luo [1], Lanfang Hu [1], Liping Guan [1] and Guangying Chen [2,*]

[1] School of Food and Pharmacy, Zhejiang Ocean University, Zhoushan 316000, China; x001022y@126.com (X.W.); 15935734529@163.com (P.D.); 15968598177@163.com (H.L.); 19818028379@163.com (L.H.); glp730@zjou.edu.cn (L.G.)

[2] Key Laboratory of Tropical Medicinal Resource Chemistry of Ministry of Education, College of Chemistry and Chemical Engineering, Hainan Normal University, Haikou 571158, China

* Correspondence: 2022150@zjou.edu.cn (B.Z.) chgying123@163.com (G.C.)

† These authors contributed equally to this work.

Abstract: Polysaccharides are very promising molecules in the field of pharmacotherapy. Knowing this, the aim of this study was to extract, characterize and evaluate the action of the polysaccharide of *noni juice* in biological models of Type 2 diabetes mellitus processes. In this study, one polysaccharide, named NJSPd-1 was separated from the fermented noni fruit juice. Characterization assay showed that NJSPd-1 had the molecular weight (*Mw*) of 18545 Da. NJSPd-1 consisted of galacturonic acid, galactose, rhamnose, and arabinose, with a molar ratio of 28.79:20.34:19.80:18.84 in HPGPC analysis, and the glycosidic bond mainly included →4)-*α*-D-GalA*p*-(1→, 4)-*β*-D-Glc*p*-(1→, →2)-*α*-L-Rha*p*-(1→, and →3)-*α*-L-Ara*f*-(1→. The prevention of oxidative stress activities of NJSPd-1 was evaluated by high glucose induced oxidative stress in HepG2 cells. In vitro result showed that, NJSPd-1 influenced the down-regulation of the proteins and genes Nrf2, Keap1, HO-1, and NQO1 in HepG2 cells. The results suggest that NJSPd-1 exerted a protective effect against oxidative stress by activating the Nrf2/HO-1/NQO1 signaling pathway in HepG2 cells.

Keywords: *Noni juice*; Polysaccharides; Structural characterization; Oxidative stress; Signaling pathway

List of Supplementary Material

Figure S1. ^{1}H NMR spectrum of NJSPd-1

Figure S2. ^{13}C NMR spectrum of NJSPd-1

Figure S3. HSQC spectrum of NJSPd-1

Figure S4. ^{1}H-^{1}H COSY spectrum of NJSPd-1

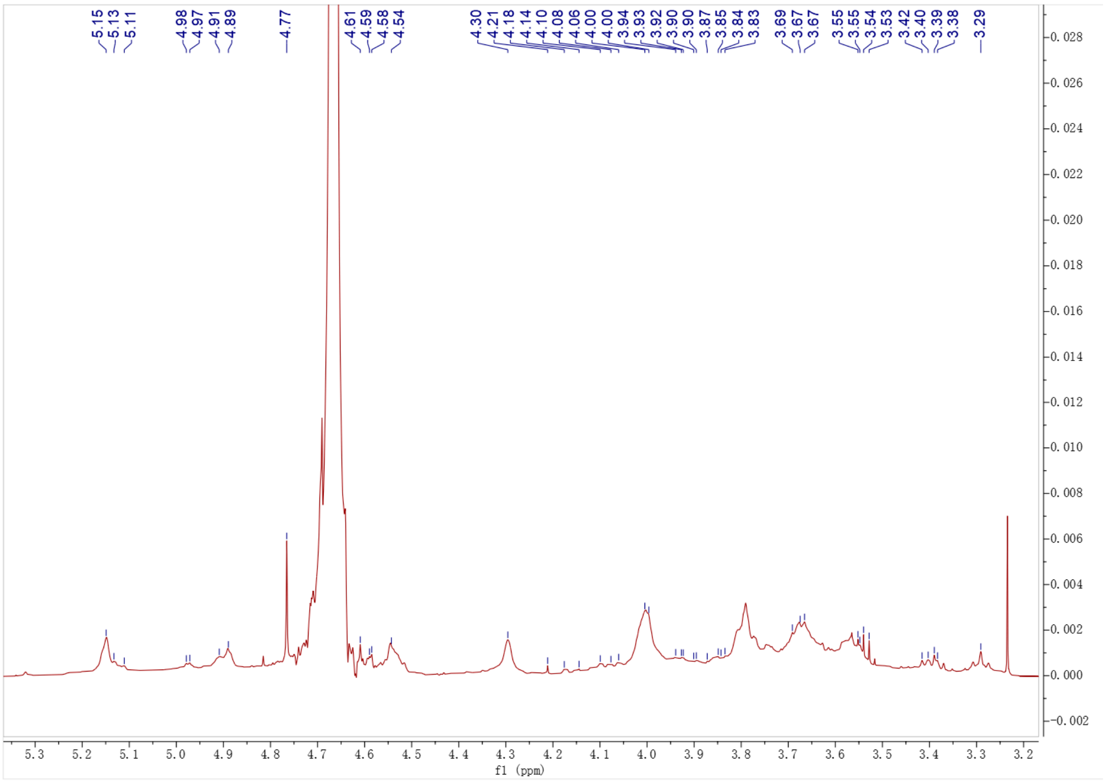

Figure S1. ^{1}H-NMR spectrum of NJSPd-1

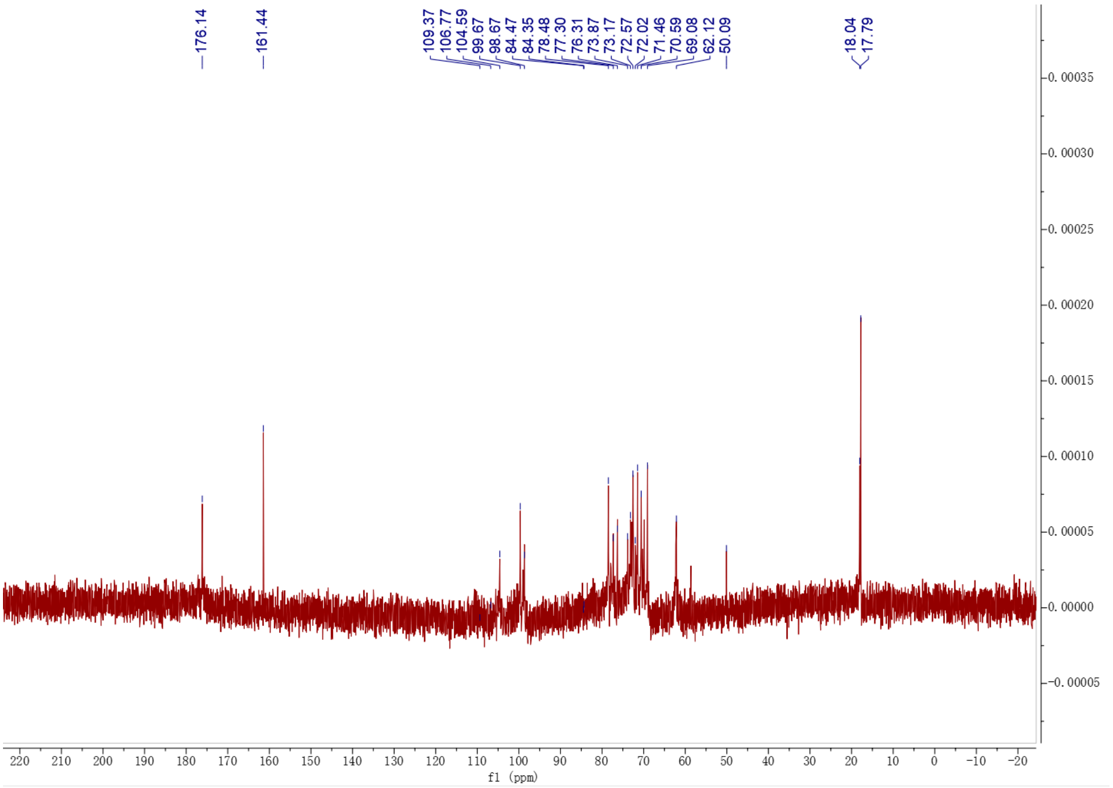

Figure S2. ^{13}C-NMR spectrum of NJSPd-1

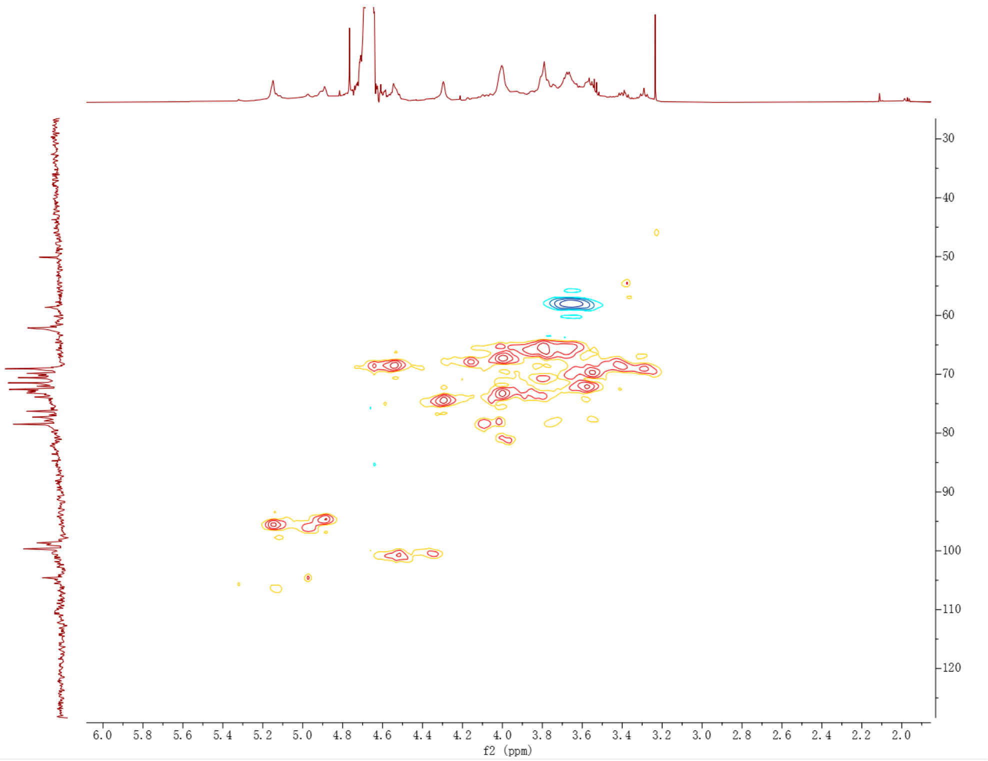

Figure S3. HSQC spectrum of NJSPd-1

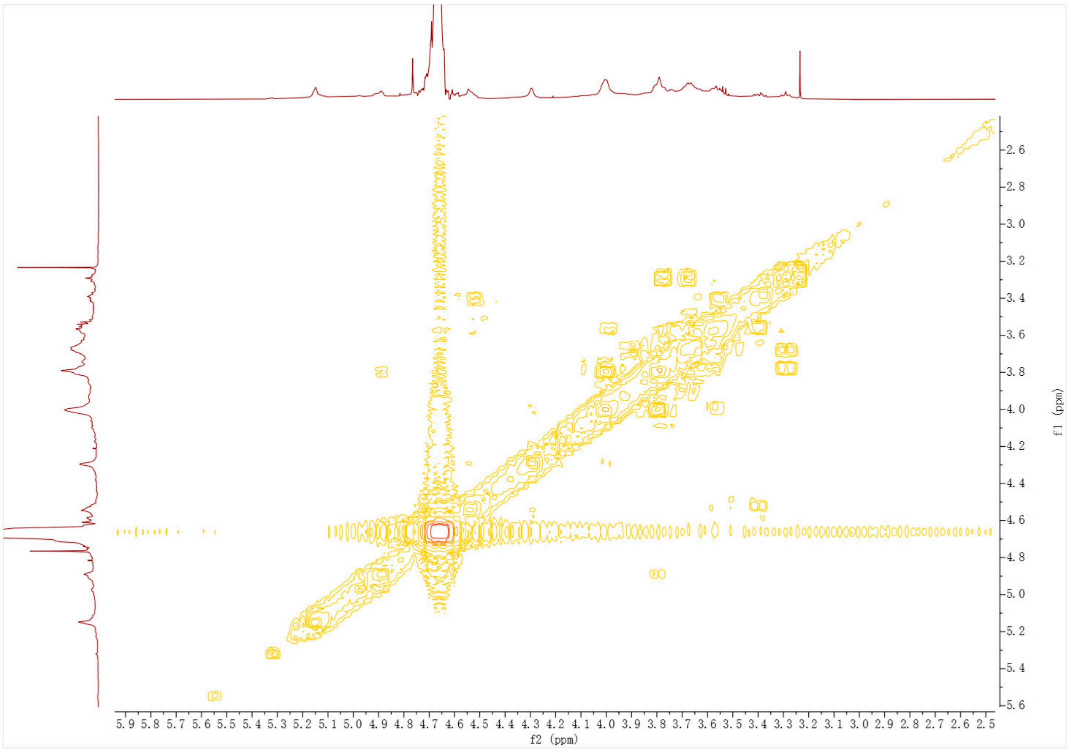

Figure S4. ^{1}H-^{1}H COSY spectrum of NJSPd-1
